# Fabrication of Cross-Sinusoidal Anti-Reflection Nanostructure on a Glass Substrate Using Imperfect Glass Imprinting with a Nano-Pin Array Vitreous Carbon Stamp

**DOI:** 10.3390/mi11020136

**Published:** 2020-01-25

**Authors:** Muhammad Refatul Haq, Jun Kim, Jeong-woo Yeom, Saem Ryu, Md. Ali Asgar, Young Kyu Kim, Seok-min Kim

**Affiliations:** Department of Mechanical Engineering, Chung-Ang University, Heukseok-dong, Dongjak-gu, Seoul 06974, Korea; refat@cau.ac.kr (M.R.H.); zuhn@cau.ac.kr (J.K.); papillon14@cau.ac.kr (J.-w.Y.); toa0125@cau.ac.kr (S.R.); asgar@cau.ac.kr (M.A.A.); kykdes@cau.ac.kr (Y.K.K.)

**Keywords:** anti-reflection nanostructure, vitreous carbon stamp, glass imprinting, imperfect imprinting, cross-sinusoidal nanostructure, glass nanostructure

## Abstract

Although polymer nanoimprinting on glass substrates has been widely employed for the fabrication of functional anti-reflective (AR) nanostructures, several drawbacks exist with respect to durability and delamination. The direct patterning of glass material is a potential solution for outdoor applications that require AR functional nanostructured glass plates. In this study, a glass imprinting technique was employed for the fabrication of an AR nanostructure on a soda-lime glass substrate using a vitreous carbon (VC) stamp. The VC stamp, which had a high aspect ratio nanopost array with a pitch of 325 nm, diameter of 110 nm, and height of ~220 nm, was fabricated by the carbonization of a replicated Furan precursor from an Si master. During the glass imprinting process using the nanopost array VC stamp, the softened glass material gradually protruded into the spaces between the nanopins owing to viscoelastic behavior, and one can achieve a cross-sinusoidal surface relief under specific imprinting condition, which can be used as an AR nanostructure with a gradually increasing refractive index. The effects of the processing temperature on the surface profile of the glass imprinted parts and the measured transmission spectra were analyzed, and a glass imprinting temperature of 700 °C and pressure of 1 MPa were found to be the optimum condition. The height of the fabricated cross-sinusoidal nanostructure was 80 nm, and the light transmission was increased by ~2% over the entire visible-light range. Furthermore, the measured transmission spectrum observed to be in good agreement with the simulation results.

## 1. Introduction

Anti-reflective (AR) functional nanostructures have been extensively researched for the suppression of the Fresnel reflection of cover glasses in solar panels and display devices [1,2]. The initial research on AR nanostructures was based on moth and butterfly eyes [3], which exhibit gradual changes in the refractive index due to the subwavelength nanostructure. A polymer nanoimprinting technique has received significant research attention with respect to the fabrication of AR nanostructures on glass substrates, due to its high replication quality at the nanoscale level and high efficiency. Lim et al. [4] fabricated a nanodome-shaped AR nanostructure on a glass substrate using ultra-violet (UV) nanoimprinting, and Chuang et al. [5] fabricated an AR nanostructure on a glass substrate using thermal nanoimprinting with an anodic alumina oxide template. Although the polymer nanoimprinting is an alternative promising method for the fabrication of AR structures on glass substrates, there are several drawbacks with respect to durability and delamination. The ideal fabrication method of AR nanostructures on glass substrates is the direct patterning of nanostructures on glass. To directly pattern an AR nanostructure on a glass substrate, reactive ion etching (RIE) with a nanoscale barrier pattern is employed. Son et. al [6] and Verma et. al [7] successfully fabricated an AR nanostructure on a glass substrate using an annealed metallic nanodot array barrier, whereas Ji et al. [8] and Yu et al. [9] employed a colloidal nanosphere; both based on RIE. Although the ideal cone-shaped AR nanostructure can be obtained using RIE with a nanoscale barrier, the RIE of glass material is time-consuming and expensive due to the low etching ratio of glass material and the toxicity of glass etching gas.

This paper proposes a glass imprinting process as an alternative cost-effective fabrication technique for uniform glass AR nanostructures. In the glass imprinting process, the glass is pressed by a stamp at a temperature higher than the glass softening temperature, to replicate the pattern on the glass [10]. Given that the glass imprinting process is conducted at high processing temperatures and pressures, a mold material with a high hardness under the processing conditions is essentially required. Among the various mold materials reported for glass nanoimprinting, vitreous carbon (VC) is regarded as the most suitable material, because it offers a high operating temperature (2500 °C in the non-oxidizing environment), chemical stability, and gas impermeability. Moreover, it provides superior releasing characteristic during the glass imprinting process [11]. Several studies were reported on the fabrication of VC stamps with nanostructures using focused ion beam milling [12], RIE with electron beam lithographed patterns, [13,14] and nanosphere lithographed patterns [15]. However, these methods are expensive when large-area VC stamps or multiple VC stamps are required. Given that the VC material is obtained by the carbonization of a high carbon yield polymer, a fabrication method for VC micro stamps was proposed based on the carbonization of a micropatterned high carbon yield polymer [16,17]. 

**Figure 1:**
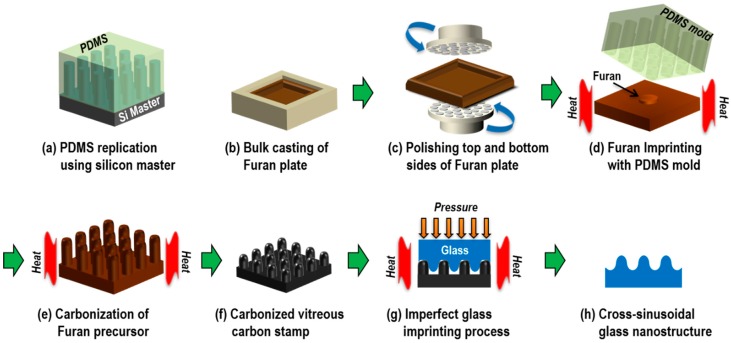
Schematic of the fabrication process for anti-reflective (AR) glass cross-sinusoidal nanostructure via imperfect glass imprinting using a high aspect ratio (HAR) nano-pin array (NPA) vitreous carbon (VC) stamp obtained by the carbonization of a Furan precursor.

A high aspect ratio (HAR) cone-shape nanostructure is preferable for the realization of a gradually changing refractive index AR nanostructure [18]. To pattern the HAR cone shape nanostructure on a glass substrate via the glass imprinting process, a stamp with an HAR negative cone shape nanostructure is required. However, it is relatively difficult to fill an HAR nanostructure via glass imprinting, as a high imprinting temperature and excessively high compression pressure are required. Hence, this paper proposes a method for the fabrication of a cross-sinusoidal nanostructure on a glass substrate via an incomplete filling technique using an HAR nanopin array (NPA) VC stamp, which is obtained by the carbonization of a replicated Furan precursor, as shown in Figure 1.

An Si master with a HAR NPA was fabricated via photolithography and RIE, and a polydimethylsiloxane (PDMS) intermediate mold was replicated from the Si master (Figure 1a). For the Furan imprinting process, a Furan substrate was obtained by bulk casting and back polishing processes (Figure 1b,c, respectively) [19]. For the fabrication of a Furan precursor with a HAR NPA, a thermal imprinting process was carried out on the Furan substrate using the PDMS intermediate mold (Figure 1d). After the carbonization process, a VC stamp with a HAR NPA was obtained. During the glass imprinting process, the softened glass material gradually protruded into the space between the NPA due to the viscoelastic behavior (Figure 1g). Hence, a cross-sinusoidal surface relief at a specific imprinting temperature and pressure was obtained, which can be considered as an AR nanostructure with a gradually changing refractive index. This paper presents an analysis of the effects of the glass imprinting conditions on the surface profile of the imprinted glass patterns, in addition to the optical transmittance characteristics of the fabricated cross-sinusoidal nanostructures, based on experimental and simulation methods.

## 2. Fabrication of Vitreous Carbon (VC) Stamp with a High Aspect Ratio (HAR) Nano-Pin Array (NPA) 

**Figure 2:**
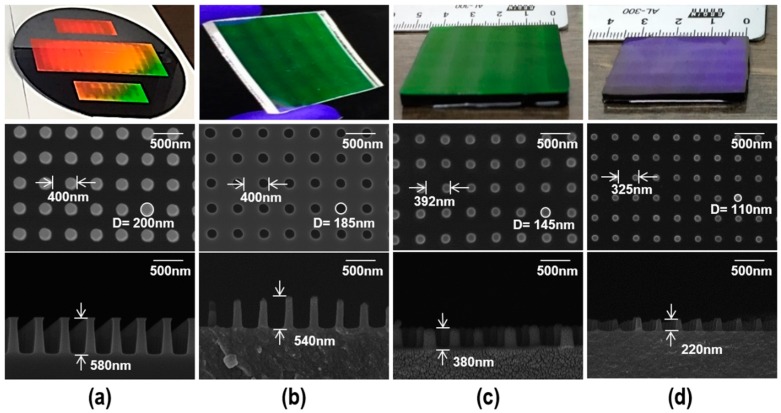
Images (top) and scanning electron microscope (SEM) images (middle: top-view, bottom: cross-sectional view) of the fabricated samples at each processing step; (**a**) Si master, (**b**) polydimethylsiloxane (PDMS) intermediate mold (the cross-sectional view image was obtained from the UV-imprinted sample from the PDMS intermediate mold), (**c**) Furan precursor, and (**d**) Vitreous carbon (VC) stamp.

To fabricate the cross-sinusoidal AR nanostructure on a glass substrate via the imperfect glass imprinting process, a VC nanostamp with a HAR NPA was fabricated by the carbonization of an imprinted Furan precursor, as shown in Figure 1a–f. Figure 2 presents the images (top) and scanning electron microscope (SEM) images (middle: top-view, bottom: cross-sectional view) of the fabricated samples at each processing step.

### 2.1. Fabrication of Si Master and Polydimethylsiloxane (PDMS) Intermediate Mold

An 8-inch Si master with a HAR NPA with a diameter of 200 nm, height of 580 nm, and pitch of 400 nm was fabricated via RIE using a KrF laser photolithographed barrier pattern (National NanoFab Center, Daejeon, Korea), as shown in Figure 2a. Although several undercut regions were observed at the top of the nanopin structure, as shown in the cross-sectional SEM image of the Si master, the HAR NPA was successfully fabricated on the entire 8-inch Si wafer via a stitching method. The cross-sectional SEM image of Si master in Figure 2a seems a line pattern due to the uniform and well-aligned NPA which was fabricated on the Si master (the defocused behind nanopin shape makes the pattern seems to line pattern). 

Given that the HAR NPA on the Si master exhibited several undercut issues, and the Si master can be easily broken during the replication process of a hard polymer material; a PDMS intermediate mold was employed in this study because the soft PDMS material can prevent damages to the Si master with the undercut structure during the replication process. To prevent adhesion during the PDMS replication process, a self-assembled monolayer (SAM) was applied onto the Si master as an anti-adhesion layer around 30 mins to lower the surface energy by immersing the Si master in 2% solution of dimethyldichlorosilane dissolved in Octamethylcyclooctasilane (Repel-Silane ES, GE Healthcare Co. Ltd., Chicago, IL, USA). 

In the PDMS replication process for the HAR NPA, it is relatively difficult to obtain a high replication quality under the conventional PDMS curing conditions, because the viscosity of uncured PDMS is not sufficiently low to fill the HAR NPA. To decrease the viscosity of the uncured PDMS and increase the replication quality, the conventional PDMS mixture (10:1 ratio mixture of Sylgard 184A and 184B, Dow Corning Co. Ltd., Midland, MI, USA) was diluted using a solvent. Thereafter, 60 wt% toluene was mixed with the conventional PDMS mixture and poured onto the Si master, thus forming a coat with a thickness of ~1 mm [20]. After the de-gassing process in a vacuum oven under 10^−1^ torr for 1 h, the PDMS mold was cured at 60 °C for 3 h. Given that the diluted PDMS mixtures resulted in fragility and poor flexibility, a conventional PDMS mixture base (mixing ratio 10:1) with a thickness of ~4 mm was poured on the already cured thin layer and cured at 60 °C for 9 h. After the second curing process, the PDMS mold with a thickness of ~5 mm was carefully peeled-off from the master and cut to dimensions size of ~50 mm × 50 mm for the following Furan imprinting process. 

As can be seen from the top-view SEM image of the PDMS mold in Figure 2b, the measured pitch was the same as that of the Si master. It clearly shows the shrinkage during the PDMS replication is negligible. However, the measured diameter of the nanohole on the PDMS mold was 185 nm, which was slightly smaller than the diameter of the nanopin on the Si master (200 nm). This difference may be due to the errors occurring in SEM measurement. For the SEM measurement, a Pt layer with a thickness of ~ 10 nm was coated onto the Si master and PDMS mold. During the Pt layer deposition, the diameter of nanopin increased slightly and that of nanohole decreased due to the side-wall deposition effects. In addition, the swelling of PDMS material in the focused area of the electron beam decreased the size of nanohole. To check the cross-sectional shape of the PDMS mold, a UV-imprinting process was carried out on an Si substrate using the PDMS mold, and the cross-sectional SEM image of the UV-imprinted pattern was obtained (bottom of Figure 2b), because the precise cutting of the softened PDMS mold was very difficult. As can be seen from the cross-sectional SEM image of the UV-imprinted nanopin pattern from the PDMS mold, the depth of the PDMS nanohole was ~540 nm (maximum height of the irregular nanopins). The irregularity of the heights of the nanopins on the UV-imprinted pattern may be due to the cutting defect. Moreover, the slight difference between the PDMS nanohole depth and Si nanopin height was due to the insufficient filling that occurred in the PDMS replication and UV-imprinting processes.

### 2.2. Fabrication of Furan Precursor with HAR NPA 

For the replication of the Furan precursor, the Furan imprinting method was applied to a Furan substrate to minimize the defects and processing time [19]. To fabricate Furan substrate, a mixture of Furan resin (KC-5302, Kangnam Chemical Inc., Seoul, Republic of Korea), ethanol (Ethyl alcohol 99%; Duksan Co. Ltd., Ansan, Republic of Korea) and p-toluenesulfonic acid monohydrate (PTSA; Kanto Chemical Co. Inc., Tokyo, Japan) with a mixing ratio of 89.9:10:0.4 wt% was poured with a thickness of 8 mm into a 50 mm × 50 mm size PDMS template without patterns and degassing process was conducted at room temperature for 1 h in a vacuum chamber. Then, the furan was cured up to 100 °C for 12 h. The Furan plate was obtained by the polishing process on both sides. For the Furan imprinting process, several drops of the Furan mixture were placed on a flat Furan plate and covered by a PDMS intermediate mold with nanoholes. Prior to Furan imprinting, the de-gassing process of the Furan mixture was conducted in a vacuum chamber to remove the air bubbles created during the mixing process. The curing process was conducted on a hot plate within the temperature range of 60–150 °C, and the temperature was maintained for 30 min and increased in 10 °C increments. After the Furan imprinting process, the imprinted Furan precursor was separated from the PDMS mold. Figure 2c presents the Furan imprinted precursor with a pitch of 392 nm, diameter of 145 nm, and height of 380 nm. Although the difference in pitch between the Furan precursor and Si master was negligible, relatively large changes in diameter (~28%) and height (~35%) were observed. The dimensional changes of the Furan precursor attributed that the Furan resin did not completely fill the HAR nanohole PDMS mold due to the high viscosity of the initial Furan resin and the shrinkage during the Furan imprinting process.

### 2.3. Carbonization of VC Stamp with HAR NPA 

To obtain the VC stamp with an HAR NPA, the carbonization of the Furan imprinted precursor was carried out in an N_2_-purged (500 cc/min) tube furnace (modified MIR-TB600-H2, Mirfurnace Co. Ltd., Pocheon, Republic of Korea) with a maximum temperature of 1000 °C. To prevent defects in the VC stamp such as warpage and micro/nano air voids during the carbonization process, the heating rates were set at 0.17 °C/min up to 600 °C and 0.33 °C/min up to 1000 °C, and then maintained at the maximum temperature for 10 h. After naturally cooled to room temperature, the VC stamp with a HAR NPA was obtained. Figure 2d presents the fabricated VC stamp with a HAR NPA with a pitch of 325 nm, diameter of 110 nm, and height of 220 nm. Although a significant shrinkage occurred in the carbonization process due to the thermal decomposition process, the pitch shrinkage had a positive influence on the AR; and the remaining NPA had an aspect ratio of 2, which is sufficient for the following imperfect glass imprinting process.

## 3. Imperfect Glass Imprinting Process for Cross-Sinusoidal Anti-Reflective Glass Nanostructure 

**Figure 3:**
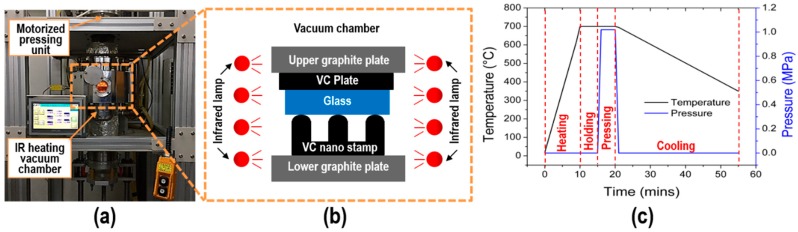
(**a**) Image and (**b**) schematics of glass imprinting system, in addition to set-up and (**c**) pressure and temperature histories of the glass imprinting process.

**Figure 4:**
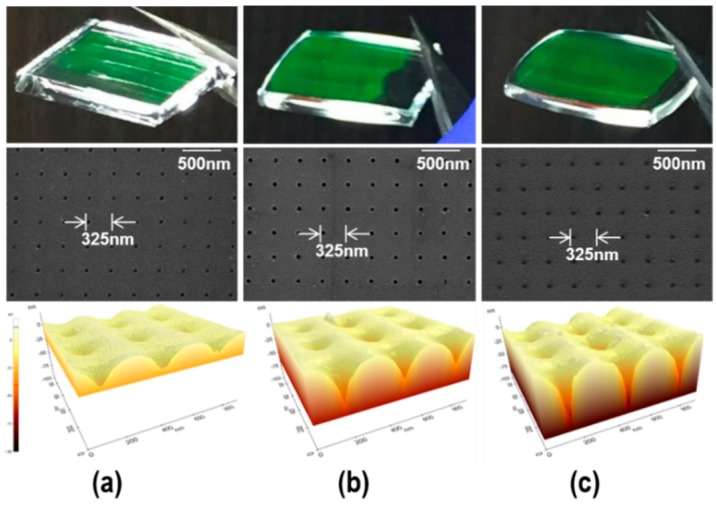
Images (top), top-view SEM images (middle), and 3D surface profiles obtained by AFM measurements (bottom) of glass imprinted samples at the imprinting temperatures of (**a**) 680 °C, (**b**) 690 °C, and (**c**) 700 °C with a fixed pressure of 1 MPa.

The glass imprinting system consists of a vacuum facilitated infrared (IR) heating chamber for a maximum operating temperature of 1,050 °C, with a temperature increase rate of 70 °C/min. In addition, a motorized pressing unit with a maximum applied force of 120 kgf was designed and constructed, as shown in Figure 3a. A low iron soda-lime glass (White Clear Glass; JMC Glass Inc., Seoul, Republic of Korea) with a glass transition temperature of 564 °C and dimensions of 20 mm × 20 mm × 3.1 mm was used as an imprinting material. Figure 3b presents the schematics of the experimental set-up for glass imprinting. The fabricated HAR NPA VC stamp was placed on the lower graphite plate, and the glass plate was located on it. An optically polished VC substrate was also placed on the glass plate to prevent damages to the backside of the glass plate due to the porous graphite plate. Figure 4c presents the typical temperature and pressure history of the glass imprinting process. When the stack of the VC stamps and glass was located on the graphite plate, an evacuation process was carried out to prevent oxidation during the glass imprinting process. Thereafter, the chamber temperature was increased to 700 °C for 10 min, and then maintained for 5 min to provide uniform temperature distribution. After the holding time, a compression pressure was applied to the stack of the VC stamp and glass substrate for 5 min. In the cooling stage, the compression pressure was released, and natural cooling was applied. After the cooling process, the glass imprinted sample was extracted to check the replication quality. 

During the glass imprinting process with the HAR NPA VC stamp, the softened glass material gradually protruded into the spaces between the nanoposts due to the viscoelastic behavior of glass material, and a cross-sinusoidal nanostructure was formed on the surface of the glass when the glass did not completely fill the nanocavity of the VC stamp. Given that the height of the cross-sinusoidal nanostructure (protrusion depth) is dependent on the imprinting temperature and pressure, and the HAR cross-sinusoidal nanostructure can provide a better AR performance; the imprinting temperature and pressure should be optimized to maximize the height of the pattern and prevent complete filling.

**Figure 5:**
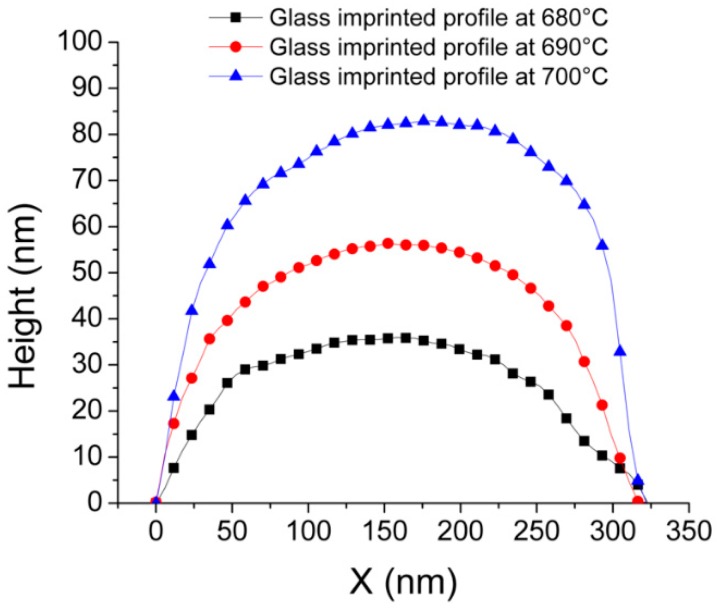
Comparison of cross-sectional surface profiles of glass imprinted cross-sinusoidal nanostructures at different imprinting temperatures.

In this study, the influence of the imprinting temperature on the height of the glass imprinted cross-sinusoidal AR nanostructure was examined at a fixed imprinting pressure of 1 MPa. It should be noted that the HAR NPA on the VC stamp can be easily damaged during the glass imprinting process due to the low structural strength of the HAR NPA. After the preliminary glass imprinting experiments with the HAR NPA VC stamp, the imprinting pressure of this study was fixed at 1 MPa, which is the maximum allowable pressure for the prevention of damages to the HAR NPA on the VC stamp. Figure 4 presents the images (top), top-view SEM images (middle), and three-dimensional (3D) surface profiles obtained by atomic force microscope (AFM) measurements (bottom) of the cross-sinusoidal nanostructured glass substrates, which were glass imprinted at temperatures of (a) 680 °C, (b) 690 °C, and (c) 700 °C; with a fixed pressure of 1 MPa. The uniform diffraction color of the samples in the images clearly indicate that the uniform nanostructures were entirely fabricated on a glass substrate without defects. In the top-view SEM images, the uniform-stamped patterns with a pitch of 325 nm were observed. The 3D surface profiles obtained by the AFM measurements indicated that the cross-sinusoidal structures were fabricated via the imperfect glass imprinting technique. To qualitatively examine the influence of the imprinting temperature on the height of the cross-sinusoidal structures, the cross-sectional surface profiles of the cross-sinusoidal structures were compared, as shown in Figure 5. The measured heights of the cross-sinusoidal nanostructures were 35 nm, 55 nm, and 80 nm for the imprinting temperatures of 680 °C, 690 °C, and 700 °C, respectively. The heights of the glass imprinted cross-sinusoidal nanopatterns were gradually increased in accordance with an increase in temperature. Although samples with greater heights could be obtained at imprinting temperatures higher than 700 °C and pressures higher than 1 MPa, several defects were observed on the glass imprinted samples due to the broken NPA on the VC stamp during the releasing process. Therefore, the optimized imprinting condition was selected as the imprinting temperature of 700 °C and pressure of 1 MPa to fabricate the highest cross-sinusoidal nanostructure on the glass substrate without any releasing problem.

## 4. AR Characteristics of the Fabricated Glass Cross-Sinusoidal Nanostructure

To examine the AR characteristics of the fabricated glass cross-sinusoidal nanostructures, the ultraviolet–visible (UV-Vis) (wavelength range of 400–800 nm) transmission spectra were measured using a spectrophotometer (V-670 UV-Vis, Jasco Inc., Easton, MD, USA) and compared with the simulated transmission spectra. 

**Figure 6:**
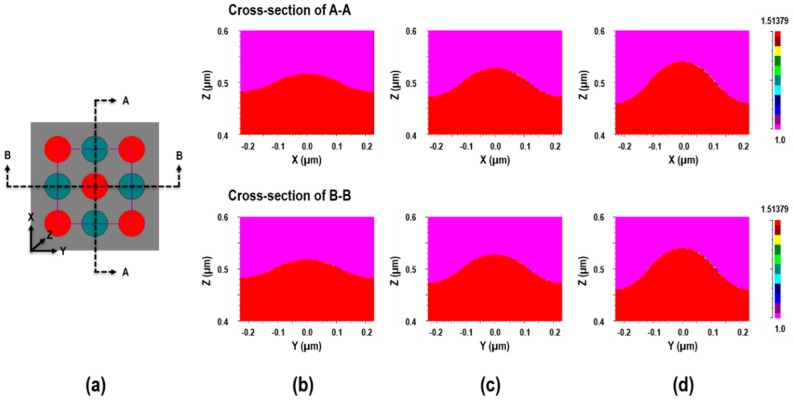
(**a**) Top-view of the 3D simulation model of the cross-sinusoidal structure generated using Rsoft computer-aided design (CAD) tool, and the A-A (top) and B-B (bottom) cross sectional contour plots of the refractive indexes of the simulation models with heights of (**b**) 35 nm, (**c**) 55 nm, and (**d**) 80 nm.

For the simulation of transmission spectra of the glass cross-sinusoidal nanostructure with different heights, a rigorous coupled wave analysis (RCWA) was conducted using commercial software (DiffractMOD, Rsoft, Synopsys, Inc., Mountain View, CA, USA). Based on the measured data of the glass imprinted cross-sinusoidal nanostructure, the 3D simulation models were constructed as shown in Figure 6. Figure 6a presents the top-view image of the 3D simulation model of the cross-sinusoidal structure, as generated using the Rsoft computer-aided design (CAD) tool. In particular, it was a combination of a positive cone structure (red), which represents glass (*n* = 1.5); and a negative cone structure (cyan), which represents air (*n* = 1), on a glass substrate (*n* = 1.5). The environmental material was set as air (*n* =1). Given that the pitch of the square-arrayed sinusoidal structure was 325 nm, and the diagonal distance between peaks was 460 nm; the x- and y-direction peak-to-valley distances (center of the positive cone and center of the negative cone) were set as 230 nm. The heights of the positive cone and depth of the negative cone were set as half of the measured heights of the cross-sinusoidal nanostructures (35 nm, 55 nm, and 80 nm). The dimensions of the simulation area were set as 460 nm × 460 nm, with repeated boundary conditions. Figure 6b−d present the A-A and B-B cross-sectional refractive index contour maps for the simulation models with total heights of (b) 35 nm, (c) 55 nm, and (d) 80 nm, respectively. 

**Figure 7:**
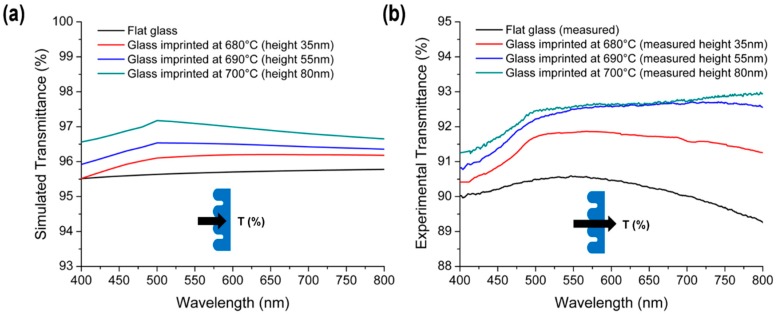
Comparison of (**a**) simulated and (**b**) measured transmission spectra of flat glass and glass imprinted cross-sinusoidal structures with imprinting temperatures of 680 °C, 690 °C, and 700 °C.

Figure 7 presents the effect of the imprinting temperature (height of cross-sinusoidal nanostructure) on the (a) simulated and (b) measured transmission spectra. In the simulation, the reflectance at the backside of the glass sample was not considered. The trends of the measured transmission results were found to be in good agreement with the simulation results. With an increase in the cross-sinusoidal nanostructure heights, the average transmittances increased in the case of the flat glass over the entire spectral range. By increasing the sinusoidal structure height from 55 nm to 80 nm, several differences were observed between the experimental transmission and simulation result, because the fabricated cross-sinusoidal structure with a height of 80 nm (Figure 4c) was not an ideal cone-shaped structure. However, both the experimental and simulation results revealed that a transmittance enhancement of ~2% in the visible wavelength was achieved from the glass imprinted cross-sinusoidal structure at a height of 80 nm, when compared with the flat glass.

## 5. Conclusions

In this study, a cross-sinusoidal structure was fabricated on a glass substrate via an imperfect glass imprinting method with a HAR NPA VC stamp. The VC stamp was obtained by the carbonization of a replicated Furan precursor, which was obtained by Furan imprinting on a Furan plate with a-PDMS replicated mold from an Si master. The Si master with an NPA having an aspect ratio of ~3 was fabricated via photolithography and RIE processes. After the PDMS replication, Furan imprinting, and the carbonization process; the VC stamp with an NPA having an aspect ratio of ~2 was obtained due to the non-filling of the highly viscous PDMS and Furan materials, in addition the shrinkages during the Furan curing and carbonization. However, the VC NPA with an aspect ratio of 2 was sufficient for the fabrication of the designed cross-sinusoidal structure via the imperfect glass imprinting process. To optimize the glass imprinting process conditions, the effects of the imprinting temperature on the height of the imprinted cross-sinusoidal structure were analyzed, in addition to the measured and simulated transmission spectra. At the imprinting temperature of 700 °C and pressure of 1 MPa, a glass imprinted cross-sinusoidal nanostructure with a pitch of 325 nm and a height of 80 nm was obtained. The transmittance of the glass imprinted cross-sinusoidal structure with a height of 80 nm was increased by ~2% over the entire visible-light range when compared with that of the flat glass substrate. This clearly indicates that the proposed imperfect glass imprinting with HAR NPA VC stamp can be employed as an alternative method for the direct fabrication of an AR nanostructure on a glass substrate. Furthermore, the fabrication of a large-area AR cross-sinusoidal nanostructured glass substrate via a roll type glass imprinting process with roll type VC mold with HAR NPA is the objective of future research.
